# New search guidance for Campbell systematic reviews

**DOI:** 10.1002/cl2.70005

**Published:** 2024-11-04

**Authors:** Heather MacDonald, Sarah Young

**Affiliations:** ^1^ Carleton University MacOdrum Library Ottawa Ontario Canada; ^2^ Carnegie Mellon University Libraries Pittsburgh Pennsylvania USA

Searching for studies in systematic reviews is a critical step that lays the foundation for the remaining stages of the review and synthesis. Searching in the social sciences and other disciplines covered by the Campbell Collaboration comes with added complexities and challenges related to finding and organizing evidence across a rich diversity of sources. To assist Campbell authors and information specialists supporting Campbell reviews in this process, we recently published new guidance (MacDonald et al., 2024) based on the previous guidance document originally published in 2010 and updated in 2017. The guide was revised to reflect current Campbell Collaboration areas of practice and recommendations in the recently updated Methodological Expectations of Campbell Collaboration Intervention Reviews (MECCIR) (Dewidar et al., 2024), capture evolving practice and strategies for searching, and update links and descriptions of individual bibliographic and other resources. It includes helpful templates, lists, and checklists to assist authors in meeting the expectations for conduct and reporting of Campbell systematic review searches. Here, we provide an overview and highlight some of the key changes and new additions.

The new guidance includes several new sections. The *Section 1.0 About this Guide* describes who this guide is for: both review authors and information specialists. Also new is the section *2.0 Working with an Information Specialist* which explains the role of the information specialist in the systematic review process. Searching for and retrieving information is a key component of systematic reviews and information specialists, as experts in search, can play a supporting or collaborative role in the production of these reviews.

In the section on *4.0 Sources to Search*, the list of sources has been placed in an Appendix which can be found on the Open Science Framework (OSF). The list can now be updated frequently so that accurate and up‐to‐date information is available to researchers. As well in this edition preprint repositories have been added to the list of potential sources of studies.

*5.0 Planning the Search* has a new section on using seed articles, or benchmarking studies, to help in the construction and validation of the search strategy. Using a seed article set can help identify search terms and ensure the search strategy finds relevant studies. Also new to the *5.3 Search updates* subsection, is the practice of checking for retracted studies. While the guidance on how to deal with retracted studies is still under debate (Faggion, 2019), checking for retractions, corrections, errata and other areas of concern related to included studies should be a routine step in any review.

The author team updated the *6.0 Designing Search Strategies* section with a new subsection on identifying search terms (both controlled vocabulary and keywords) and how to use text mining for selecting terms. Inclusion of a discussion on predatory publications is also new providing guidance on deciding how to deal with potential predatory publications. The subsection *6.5.7 Adapting search strategies across databases* is another addition in this version of the guide complete with examples. The subsection *6.6*, previously called *Additional strategies*, has been updated and renamed *Supplementary search techniques* to be in keeping with the TARCiS statement by Hirt et al. (2023). A new subsection *6.8 Peer review of search strategies* on search peer reviews has been added. Peer review of search strategies occurs during standard peer review processes. However, search strategies are complex, and minor typos or syntax errors can have drastic implications for search results and thus review findings. For this reason, it is recommended that the search, in particular, be peer reviewed before manuscripts are submitted as an added checkpoint. We have also added a section *6.9 When to stop searching*. In searching for studies in the social sciences, especially when included study designs are diverse and much of the research may be found in grey literature, identifying when ‘enough is enough’ can be particularly challenging. This section addresses this challenge and provides some considerations for stopping rules when it comes to searching and search strategy development.

A new section on *8.0 Selecting Studies* was added to this version of the guide, similar to the Cochrane handbook. While the selection of studies is not strictly part of the searching step of reviews, there are important information management considerations in the screening phase that the author team, as librarians and information specialists, felt would be helpful to address. The section *9.0 Documenting and Reporting the Search* was updated to include the recently released MECCIR standards (Dewidar et al., 2024) and the PRISMA‐S reporting guideline (Rethlefsen et al., 2021).

A total of five *Appendices* can be found on OSF. They include a list of databases by subject, grey literature sources by geography, documenting and reporting templates, a peer review checklist for searches, and a list of abbreviations and definitions found in the guide. We hope that researchers will find these appendices useful for their own systematic searches.

In conclusion, this new document, providing guidance along with templates and checklists, should be a go‐to resource for any new or seasoned Campbell review author. In a recent assessment of Campbell systematic reviews, we found that only about 10% of reviews published since 2017 had cited the previous Campbell searching guidance (Young et al., 2024). We hope that the updated version of the Campbell searching guidance will become a routine reference document for all Campbell authors moving forward. We also encourage authors new to conducting systematic review searches in the social sciences to take the Campbell Collaboration's online course on systematic reviews and meta‐analysis (Unit 3 covers searching and is an excellent companion resource to the search guidance), which, as of the writing of this editorial, is freely available through the Open Learning Initiative (Valentine et al., 2022). With the support of these resources, and by involving a trained information specialist, researchers will be well equipped to produce thorough, robust, and transparent searches to support high‐quality evidence synthesis and contribute to building a credible and trustworthy evidence base.
